# Supplementary material for: Plasma C-Peptide and Risk of Developing Type 2 Diabetes in the General Population
Source: J Clin Med. 2020 Sep 17;9(9):3001. doi: 10.3390/jcm9093001 (PMC7564789; doi:10.3390/jcm9093001)
Supplement: Supplementary file 1 [file jcm-09-03001-s001.pdf]

Table S1. Multivariable adjusted linear regression analyses with log-transformed C-peptide as dependent variable in 5176 participants of the PREVEND study.

Variables	Age- and sex-adjusted		Age-, sex-, and insulin-adjusted		Multivariable adjusted (R^2=0.721)	
	Standardized β	*p* value	Standardized ß	*p* value	Standardized ß	*p* value
Sex, female vs. male	—	—	—	—	0.040	<0.001
Age, years	—	—	—	—	…	…
Family history of diabetes, yes vs. no	0.071	<0.001	0.033	<0.001	…	…
Alcohol, yes vs. no	-0.09	<0.001	…	…	…	…
BMI, kg/m²	0.530	<0.001	0.179	<0.001	0.120	<0.001
Systolic blood pressure, mm Hg	0.235	<0.001	0.060	<0.001	-0.039	0.002
Dystolic blood pressure, mm Hg	0.207	<0.001	0.072	<0.001	0.056	<0.001
Antihypertensive medication, yes vs. no	0.202	<0.001	0.07	<0.001	…	…
Total cholesterol, mmol/L	0.108	<0.001	0.023	<0.001	…	…
HDL cholesterol, mmol/L	-0.416	<0.001	-0.143	<0.001	-0.052	<0.001
Triglycerides, mmol/L	0.458	<0.001	0.181	<0.001	0.140	<0.001
Lipid-lowering medication, yes vs. no	0.132	<0.001	0.046	<0.001	…	…
Plasma ASAT (U/L)	0.150	<0.001	…	…	-0.070	<0.001
Plasma ALAT (U/L)	0.333	<0.001	0.084	<0.001	0.103	<0.001
Glucose, mmol/L	0.336	<0.001	0.113	<0.001	0.096	<0.001
Insulin (Mu/L)	0.754	<0.001	—	—	0.549	<0.001
eGFR, mL/min/1.73 m^2	-0.275	<0.001	-0.162	<0.001	-0.151	<0.001
Urinary albumin excretion, mg/24 hours	0.161	<0.001	0.061	<0.001	0.029	0.001

ASAT: L-aspartate aminotransferase; ALAT: L-alanine aminotransferase; eGFR: estimated glomerular filtration rate; PREVEND: Prevention of renal and vascular End-stage Disease

Table S2. Additive Value of C-Peptide and Insulin, for the Prediction of Type 2 Diabetes

	C-statistics (95% CI)	*p* value for change in C-statistics
FOS risk model	0.888 (0.870-0.907)	
Fos risk model+ C-peptide	0.891 (0873-0.909)	0.04
Fos risk model+ insulin	0.889 (0.870-0.908)	0.27

FOS: Framingham Offspring risk score

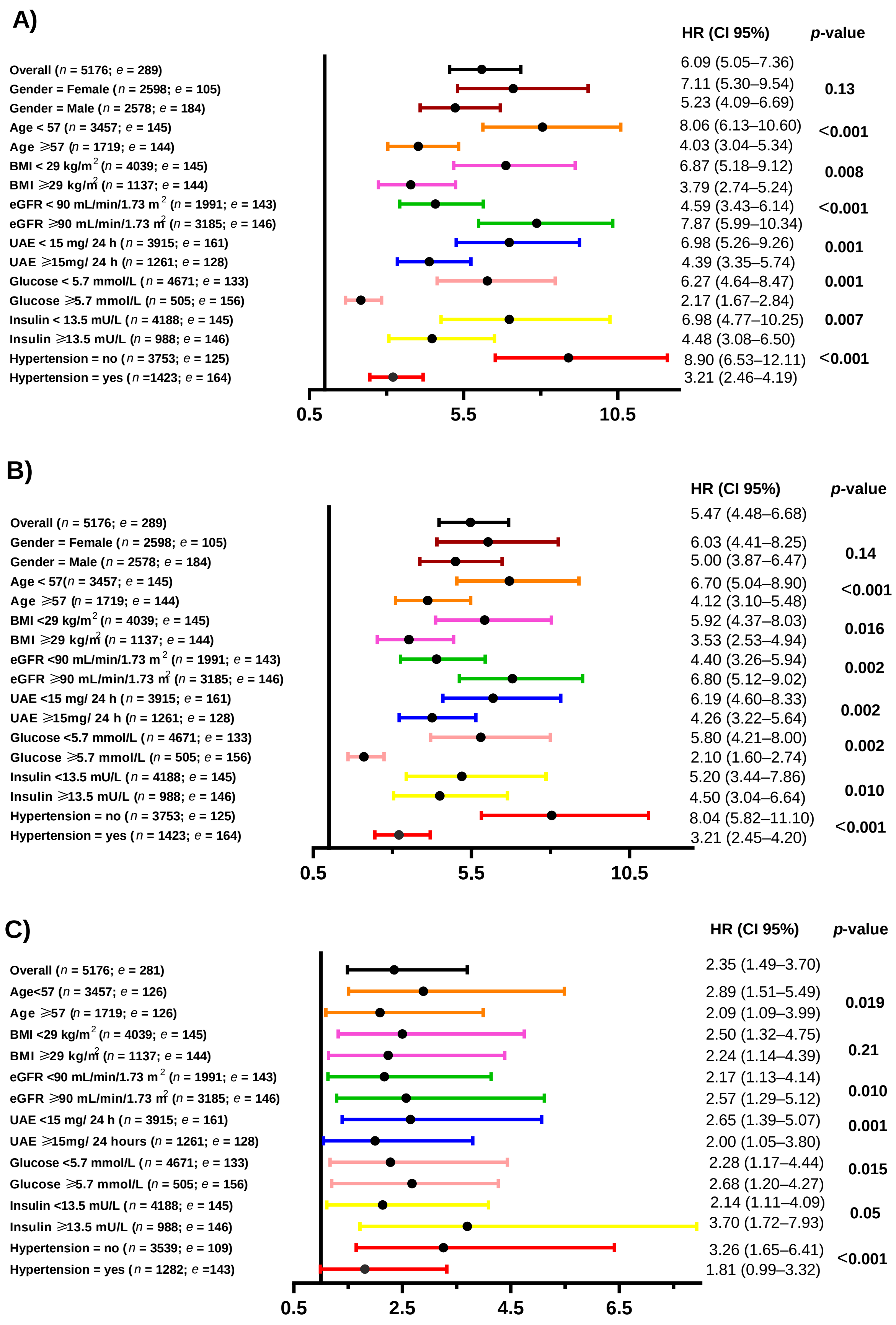

Figure S1. Association between C-peptide and risk of diabetes in the overall population and stratified by selected characteristics (*n*= number of subjects; *e* = number of cases). Multivariable hazard ratios (95% confidence

intervals) for risk of diabetes are expressed per Log_2-unit increase of C-peptide levels. Hazard ratios (95 CIs) were derived from Cox proportional hazards regression models with crude analyses in panel (**A**), adjusted for age, sex in panel (**B**), and adjusted for age, sex, smoking status, alcohol consumption, BMI, family history of diabetes, hypertension, triglycerides, total cholesterol, HDL cholesterol, eGFR, urinary albumin excretion, glucose and insulin in panel (**C**). The *p*-values denote the *p* for interaction. BMI: body mass index; HDL: high-density lipoprotein; eGFR: estimated glomerular filtration rate; UAE: urinary albumin excretion

Table S3.Competing risk model of C-peptide to mortality and incident Type 2 Diabetes in 5176 participants without diabetes.

	Subhazard Ratio (95% CI)	*p value*
Crude analysis	5.96 (4.91–7.23)	<0.001
Model1†	5.48 (4.42–6.74)	<0.001
Model2‡	5.31 (4.29–6.57)	<0.001
Model3§	3.47 (2.67–4.52)	<0.001
Model4ǁ	2.88 (2.15–3.87)	<0.001
Model5¶	3.29 (2.39–4.52)	<0.001
Model6#	2.33 (1.49–3.65)	<0.001

Subhazard Ratio (95% CIs) were derived from competing risk analyses by Fine and Gray .
†Model 1 is adjusted for age and sex.
‡Model 2 is additionally adjusted for smoking status and alcohol consumption.
§Model 3 is additionally adjusted for BMI, family history of diabetes and hypertension.
||Model 4 is additionally adjusted for triglycerides, total cholesterol and HDL cholesterol.
¶Model 5 is additionally adjusted for eGFR and urinary albumin excretion.
#Model 6 is additionally adjusted for glucose and insulin.
eGFR: estimated glomerular filtration rate; PREVEND: Prevention of renal and vascular End-stage Disease.

Table S4. Association between C-peptide and risk of developing Type 2 Diabetes in 5,176 participants of the PREVEND study*

	Sex specific tertiles of plasma C-peptide, pmol/L				C-peptide per Log_2 unit increase	*p* value
	Male	<642	642-890	>890		
	Female	<592	592-803	>803		
		1	2	3		
Cases		19	57	213	289	
Crude analysis		1.00 (ref)	3.06 (1.81-5.17)	12.61(7.84-20.26)	7.10 (5.63-8.94)	<0.001
Model1†		1.00 (ref)	2.91 (1.72-4.93)	11.54 (7.13-18.69)	6.60 (5.19-8.40)	<0.001
Model2‡		1.00 (ref)	2.87 (1.70-4.87)	11.21(6.92-18.16)	6.46 (5.07-8.22)	<0.001
Model3§		1.00 (ref)	2.19 (1.29-3.73)	5.55 (3.32-9.28)	4.07 (3.07-5.39)	<0.001
Model4ǁ		1.00 (ref)	2.07(1.18-3.63)	4.29 (2.45-7.51)	3.42 (2.49-4.70)	<0.001
Model5¶		1.00 (ref)	1.99(1.12-3.49)	4.09 (2.33-7.19)	3.89(2.78-5.46)	<0.001
Model6#		1.00 (ref)	1.64(0.91-2.95)	2.19 (1.15-4.18)	2.43(1.43-4.13)	0.001

*ORs (95% CIs) were derived from design-based logestic regression models.
†Model 1 is adjusted for age and sex.
‡Model 2 is additionally adjusted for smoking status and alcohol consumption.
§Model 3 is additionally adjusted for BMI, family history of diabetes and hypertension.
ǁModel 4 is additionally adjusted for triglycerides, total cholesterol and HDL cholesterol.
¶Model 5 is additionally adjusted for eGFR and urinary albumin excretion.
#Model 6 is additionally adjusted for glucose and insulin.
eGFR: estimated glomerular filtration rate; PREVEND: Prevention of renal and vascular End-stage Disease.
